# Pathogenomics and clinical recurrence influence biofilm capacity of *Escherichia coli* isolated from canine urinary tract infections

**DOI:** 10.1371/journal.pone.0270461

**Published:** 2022-08-25

**Authors:** Gregory A. Ballash, Dixie F. Mollenkopf, Dubraska Diaz-Campos, Joany C. van Balen, Rachel E. Cianciolo, Thomas E. Wittum

**Affiliations:** 1 Department of Veterinary Preventive Medicine, The Ohio State University, Columbus, Ohio, United States of America; 2 Department of Veterinary Clinical Sciences, The Ohio State University, Columbus, Ohio, United States of America; 3 Department of Veterinary Biosciences, The Ohio State University, Columbus, Ohio, United States of America; Cairo University, EGYPT

## Abstract

Biofilm formation enhances bacteria’s ability to colonize unique niches while protecting themselves from environmental stressors. *Escherichia coli* that colonize the urinary tract can protect themselves from the harsh bladder environment by forming biofilms. These biofilms promote persistence that can lead to chronic and recurrent urinary tract infections (UTI). While biofilm formation is frequently studied among urinary *E*. *coli*, its association with other pathogenic mechanisms and adaptations in certain host populations remains poorly understood. Here we utilized whole genome sequencing and retrospective medical record analysis to investigate associations between the population structure, phenotypic resistance, resistome, virulome, and patient demographic and clinical findings of 104 unique urinary *E*. *coli* and their capacity to form biofilms. We show that population structure including multilocus sequence typing and Clermont phylogrouping had no association with biofilm capacity. Among clinical factors, exposure to multiple antibiotics within that past 30 days and a clinical history of recurrent UTIs were positively associated with biofilm formation. In contrast, phenotypic antimicrobial reduced susceptibility and corresponding acquired resistance genes were negatively associated with biofilm formation. While biofilm formation was associated with increased virulence genes within the cumulative virulome, individual virulence genes did not influence biofilm capacity. We identified unique virulotypes among different strata of biofilm formation and associated the presence of the *tosA/R-ibeA* gene combination with moderate to strong biofilm formation. Our findings suggest that *E*. *coli* causing UTI in dogs utilize a heterogenous mixture of virulence genes to reach a biofilm phenotype, some of which may promote robust biofilm capacity. Antimicrobial use may select for two populations, non-biofilm formers that maintain an arsenal of antimicrobial resistance genes to nullify treatment and a second that forms durable biofilms to avoid therapeutic insults.

## Introduction

Extraintestinal *Escherichia coli* (ExPEC) are a group of pathogenic *E*. *coli* that cause infections outside of the intestinal tract [1]. ExPEC are often classified based on the organ system they infect and the unique set of virulence genes that permits these infections. Specific pathogenic types of ExPEC include those that cause neonatal meningitis and septicemia in humans and systemic infections like avian pathogenic *E*. *coli* in veterinary species [1, 2]. ExPEC that can colonize the urinary tract, commonly referred to as uropathogenic *E*. *coli* (UPEC), are the leading cause of urinary tract infections (UTIs) in humans and dogs [3]. UTIs are among the most common reasons for dogs to see their primary care veterinarian, affecting nearly 1 in 6 dogs in their lifetime [4]. While most of these infections are sporadic, UPEC UTI can recur in up to 20% of dogs [5, 6]. In addition, antimicrobial resistance (AMR), including multidrug resistance and resistance to last lines of therapy, is an emerging and increasing concern among UPEC UTI in both humans and dogs [5]. Together, recurrence and AMR puts clinicians, clients, and patients in uncomfortable scenarios that include treatment failures, prolonged morbidity, client/patient frustration, and increased financial burdens [7].

Biofilms are dynamic communities of heterogeneous microorganisms that transition from a free-swimming, planktonic state to a sessile state that is embedded in an extracellular matrix [8]. Approximately 60–80% of UPEC causing UTI have been reported to be capable of forming a biofilm [9]. Biofilms can serve as hotspots for intra- and interspecies transfer of genetic material that can harbor AMR, virulence, and fitness genes that promote subpopulation diversity and resistance to antimicrobial treatment and environmental stressors. These adaptations can promote persistence in environmental niches and are associated with recurrent and chronic UPEC UTI [10].

Biofilm formation is initiated by bacterial attachment followed by progression to microcolony formation and biofilm maturation [11]. Several studies identified key virulence genes associated with biofilm formation in mouse models of UPEC UTI including flagella, adhesins, siderophores and autotransporters. However, the significance of these genes in biofilm formation is not always reproducible across observational studies of infecting UPEC strains. Virulence gene redundancy is common in UPEC strains and this redundancy can rescue biofilm phenotypes when individual biofilm-associated genes are non-functional [12]. These observations suggests that UPEC may mix-and-match virulence traits to express biofilm phenotypes. Similarly, certain antimicrobial resistance phenotypes and genes, particularly those that confer fluoroquinolone resistance, have been associated with biofilm formation [10, 13]. However, the relationship between AMR and biofilms is inconsistently reported across the literature and suggest that the true relationship is unknown [14, 15].

The capacity of biofilm formation of canine UPEC and its relation to virulence genes and AMR phenotypes and genotypes is not well understood. Because canine UPEC can represent different strains with different antimicrobial and virulence capacities when compared to typical human UPEC, a broad survey of canine UPEC would generate needed information on the relationship between AMR, virulence and biofilm formation. Here we describe a whole genome survey of 104 canine UPEC isolates and associate their population structure, AMR phenotype and genes, and virulence gene with biofilm capacity. In addition, we report the relationship of patient demographic and clinical characteristics with isolate’s biofilm formation.

## Materials and methods

### Sample collection and retrospective medical record review

*E*. *coli* isolates were collected from post-diagnostic cultures of canine patients with a confirmed clinical UTI. Isolates were selected to represent diagnostic *E*. *coli* recovered from UTI by the OSU Veterinary Medical Center Diagnostic and Clinical Microbiology Laboratory from 2018–2020. All isolates were collected via cystocentesis and were confirmed to be *E*. *coli* at the time of diagnosis and prior to molecular and phenotypic characterization using MALDI-TOF. Isolates were frozen in a 1:1 mixture of tryptic soy broth and 60% glycerol at -80° C until phenotypic and molecular characterization. Isolate collection for this project was determined to be exempt from IACUC review because the samples were originally collected for diagnostic purposes and only used for research following their diagnostic utility.

The medical record for each dog included in the study was reviewed for patient information including age, breed, sex/neuter status, comorbidities known or suspected to increase the risk of UTI, history of a recurrent UTI, currently in a recurrent UTI state which was defined using guidelines previously established in veterinary medicine, current use of psychotics/sedatives, analgesics, and/or immunosuppressive medication, a diagnosis or suspicion of pyelonephritis made by the clinician and diagnostic team, the presence of clinical signs indicative of a urinary tract infection and antimicrobial use within the past 30 days [16]. Comorbidities, history of recurrent UTI, currently in a recurrent UTI state, use of psychotics/sedatives, analgesics, or immunosuppressive medication, pyelonephritis, presence of clinical signs and previous antimicrobial use were collected as binary data points. Sex/neuter status and breed were categorized as intact female, spayed female, intact male and castrated male and small (<12 kg), large (> 12kg) and mixed breed. Age was collected as a continuous variable. Dogs that lacked a complete history or medical record or that could not be traced back 30 days from their initial infection using their medical record were removed from any analyses that involved this clinical feature.

### Biofilm capacity characterization

The ability of isolates to form biofilms was determined using a slightly modified version of an assay that has been previously described [17]. In brief, 20 μl of *E*. *coli* suspension adjusted to a 0.5 MacFarland standard was inoculated in 180 μl of tryptic soy broth (TSB) and allowed to aerobically grow at 37 C for 24 hours under static conditions. After incubation, the broth was removed, and each well was washed three times with sterile phosphate-buffered saline. Plates were allowed to dry at room temperature in an inverted position overnight. Wells were then stained with 200 μl of 0.1% crystal violet for 15 minutes at room temperature. Each well was washed three times with distilled water and then dried for 30 minutes in a 37 C incubator. Once completely dry, 200 μl of 96% ethanol was added and incubated for 30 minutes at room temperature. Plates were read using an Epoch microplate spectrophotometer (BioTek Instruments, Winooski, VT, USA) at an optical density of 570 nm. Biofilm formation was methodically replicated three times, with each replication consisting of four technical replicates. A positive control using *E*. *coli* ATC2273, and negative control wells consisting of 200 μl of uninoculated TSB, were included on each plate.

The twelve optical density readings were averaged to obtain a mean OD 570 reading for each strain. Biofilm formation was qualitatively graded in four categories based on OD values compared to a cut-off OD (cOD). cOD was defined as the mean OD of the negative control plus three standard deviations. The cutoffs were as follows: sample OD < cOD = non-bioiflm producer; cOD < sample OD ≤ 2*(cOD) = weak biofilm producer; 2*(cOD) < sample OD ≤ 4*(cOD) = moderate biofilm producer; sample OD > 4*(cOD) = strong biofilm producers. For the purpose of data analysis, moderate and strong biofilm producers were collapsed into a strong/moderate biofilm category.

### Antimicrobial resistance phenotype characterization

Minimum inhibitory concentrations (MIC) to a panel of antimicrobial drugs important to human and veterinary medicine were determined using the Sensititre Vizion (COMPGN1F plate; Thermo Fisher Scientific, Oakwood Village, OH, USA) broth micro-dilution system following Clinical and Laboratory Standards Institute (CLSI) guidelines [18]. MICs for 16 antibiotics–ampicillin, amoxicillin with clavulanic acid, cefazolin, cefpodoxime, ceftazidime, imipenem, chloramphenicol, tetracycline, doxycycline, enrofloxacin, pradofloxacin, marbofloxacin, gentamicin, amikacin, piperacillin tazobactam, and trimethoprim sulfamethoxazole–were used for analysis. The antibiotics were tested at predetermined dilution ranges according to the manufactured MIC plate (COMPGN1F). Isolates were classified as susceptible or reduced susceptible (RS), including those with an “intermediate” classification, using the CLSI breakpoints [18]. *E*. *coli* strains ATCC 25922 and 35218 were used as quality controls. The definition of multidrug-reduced susceptibility (MDRS) was modified from the definition of multidrug-resistance and defined as reduced susceptibility to at least one antimicrobial from three or more drug categories [19].

### Whole genome sequencing and molecular characterization

Total DNA was extracted from *E*. *coli* using the Qiagen DNeasy Blood and Tissue Kit (Qiagen Inc., Valencia, CA, USA). DNA was checked for quality and quantity using a NanoDrop 2000 (Thermo Fisher Scientific, Wilmington, DE, USA) spectrophotometer and Qubit 2.0 fluorometer (Life Technologies, USA), respectively. Sequencing libraries were prepared with the NextEra XT library preparation kit (Illumina Inc., San Diego, CA, USA). All isolates underwent short-read sequencing using the Illumina MiSeq platform. Adapter sequences were trimmed, and sequencing data were quality assessed using TrimGalore and FastQC, respectively [20, 21]. The resulting sequences were assembled using Unicycler and contigs were annotated using Prokka [22, 23]. Assembled sequences were assessed for acquired antimicrobial resistance genes using the ResFinder 4.1 online database with default parameters from the Center of Genomic Epidemiology [24]. Annotated contigs were compared to a library of UPEC-specific virulence protein sequences acquired via EcolVF [25]. Virulence genes were considered present if there was at least 80% coverage and 80% identity to the reference protein.

Whole-genome sequences were analyzed for phylogroup composition using the Clermont Phylotyper [26]. Isolates were further characterized into sequence types and sequence type complexes using the Achtman MLST criteria [27]. SNP phylogenetic analysis and genomic comparisons was conducted with CSIPhylogeny using default parameters [28]. The laboratory strain *E*. *coli* str. K12, substr. MG1655 (Genbank ID: LR881938), was used as reference sequence for SNP phylogenetic analysis. Maximum likelihood trees were visualized using the Interactive Tree of Life interface (iTOL) [29].

### Co-occurrence network analysis and virulotype identification

Co-occurrence network matrices were generated for individual levels (none, weak, moderate/strong) of phenotypic biofilm production using Spearman’s correlation with post-hoc Sidak corrected P-values. Only unique gene pairs with corrected P-value < 0.05 and a rho ≥ 0.6 were included in the network analysis. Networks were constructed and described using the Igraph package in R and virulence gene virulotypes/communities were partitioned using the fast_greedy_algorithm [30]. Final networks were visualized using Gephi [31]. Enriched virulence genotypes were analyzed for associations with biofilm phenotype using logistic regression.

### Data analysis

We utilized ordinary logistic regression models to estimate the association between biofilm formation and population structure, patient demographics and clinical history. Logistic regression models for patient demographic variables only used demographics at the time of initial urinary isolate collection to control for multiple samples collected from individual dogs. Biofilm formation was dichotomized into two groups for comparison—any biofilm (weak, moderate/strong) production and strong biofilm (moderate/strong) production. Logistic regression models were constructed using any or strong biofilm production as the outcome variable. A forward selecting model building approach was utilized by conducting univariate analysis initially. Those variables with a P-value ≤ 0.2 or that were considered as known biological or clinical confounders were included in the final multivariable model. Variables in the multivariate model were then excluded if they had a P-value > 0.05 and did not alter the remaining variables coefficients by > 20%, indicative of confounding effects. The final models were tested for parsimony using a likelihood ratio test.

Additional logistic regression models were constructed using a similar forward selection approach to test for associations between reduced phenotypic susceptibility and multidrug resistance and for differences in the proportion of individual virulence genes and resistance genes. For analysis that utilized multiple logistic regression models such as those evaluating associations between individual virulence genes and AMR genes, a post-hoc correction using the Yekutieli method was employed to calculate false discovery rates. False discovery rates at a q-value ≤ 0.10 were used to control type I error. Forward constructed Poisson regressions were utilized to identify relationships between counts of resistance and virulence genes and biofilm capacity. All statistical analysis was conducted using STATA v.15 (StataCorp LLC, College Station, TX, USA).

## Results

### Population structure is not associated with biofilm formation

**Fig 1:**
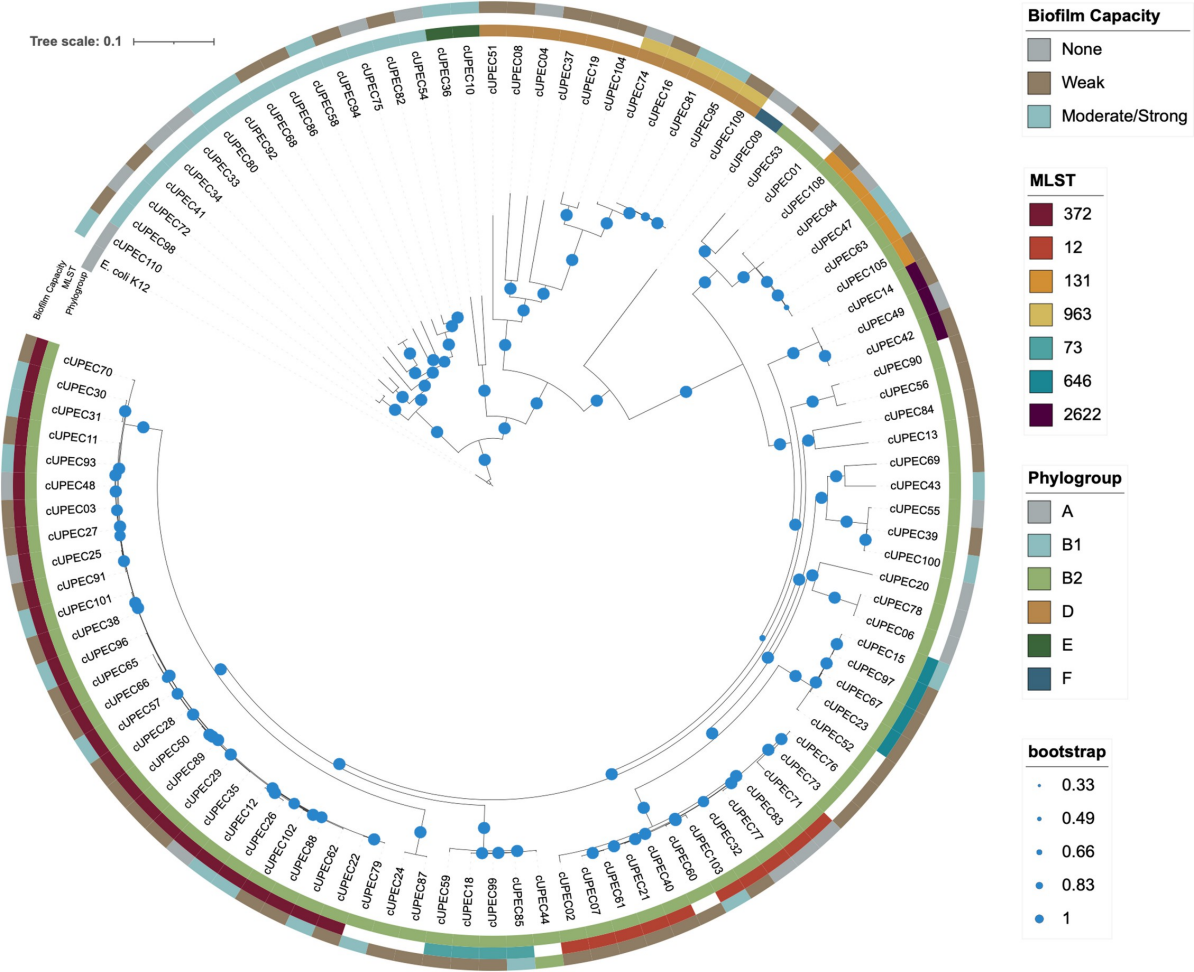
Maximum likelihood tree of 104 canine UPEC isolates. Maximum likelihood phylogenetic tree of core SNP variation among 104 canine UPEC isolates and their associated phylogroup (innermost ring), most frequent MLST sequence types (middle ring), and biofilm capacity (outermost ring). Biofilm formation was categorized (none, weak, moderate/strong) based on a qualitative scale separated by specific optical density value cut-offs.

A total of 104 unique UPEC isolates from 94 different dogs were collected and tested for their capacity to form biofilms. Of those, 20.2% (21/104) were not capable of forming a biofilm. The remainder (79.8%; 83/104) could form a biofilm with a distribution of 55.8% (58/104) forming weak biofilms and 24% (25/104) forming moderate or strong biofilms (Fig 1). Core genome SNP analysis of the phylogenetic structure of our UPEC isolates included 3,397,874 nucleotide positions covering approximately 73.5% of the reference genome. The UPEC population was composed of six different phylogroups with most isolates representing phylogroup B2 (75/104; 72%), followed by B1 (14/104; 13.5%) and D (11/104; 10.6%). Phylogroup A, E, and F were also represented, but combined for only four isolates (3.8%). Utilizing Achtman MLST, we identified 45 different sequence types. While most ST were only represented by less than three isolates, ST372 and ST12 represented 26% (27/104) and 9.6% (10/104) of our isolate population, respectively. Other ST with three or more isolates included ST131, ST2622, ST646, ST963 and ST73. These ST combined to represent 21% (22/104) of the UPEC strains. There was no difference in biofilm formation between phylogroup (any: P = 0.33; strong: P = 0.94) or MLST (any: P = 0.66; strong: P = 0.81). When all other phylogroups were compared to the typical UPEC pathogenic phylogroup, phylogroup B2, there was no association with any (P = 0.25) or strong (P = 0.60) biofilm capacity.

### UTI recurrence is associated with biofilm formation

Retrospective medical record analysis was utilized to obtain patient demographic and clinical history information and to investigate the association of these factors with the capacity to form biofilms (Table 1). Most patient epidemiologic characteristics were not associated with the capacity of urinary *E*. *coli* to form *in vitro* biofilms. However, isolates from dogs that had a history of chronic/recurrent urinary tract infections were at 8.5 times the odds of forming a biofilm (95% CI: 1.07, 67.6; P = 0.043) compared to isolates from dogs with no history of chronic UTI. Similarly, dogs that were currently in a state of recurrence were at 3.73 times the odds of having an isolate form a strong biofilm compared to dogs not actively in recurrence (95% CI: 1.37, 10.20; P = 0.01).

**Table 1:** Univariate logistic regression analysis of patient factors and biofilm formation.

	Any Biofilm Formation			Moderate/Strong Biofilm Formation		
Predictor Variables	OR	95% CI	P-value	OR	95% CI	P-value
Patient Demographics						
Age	0.89	0.77, 1.02	0.088	1.02	0.91, 1.14	0.749
Sex			0.71			0.81
*Spayed Female*	Referent	.	.	Referent	.	.
*Intact Female*	2.6	0.30, 22.19	0.382	0.6	0.12, 3.07	0.541
*Castrated Male*	0.715	0.20, 2.62	0.612	0.98	0.28 3.51	0.98
*Intact Male*	1.04	0.11, 10.11	0.13	.	.	.
Breed			0.13			0.88
*Small*	Referent	.	.	Referent	.	.
*Large*	1.69	0.40, 7.12	0.472	1.17	0.35, 3.97	0.797
*Mixed*	0.52	0.14, 1.94	0.329	0.9	0.24, 3.31	0.869
Therapeutic History						
Short-term Antimicrobial Exposure (<3 days)	0.52	0.18, 1.47	0.217	0.38	0.10, 1.39	0.143
Long-term Antimicrobial Exposure (<30 days)	0.94	0.34, 2.66	0.914	0.94	0.35, 2.50	0.896
>1 Antimicrobial Past 30 days	1.91	0.34, 10.68	0.462	4.69	1.05, 20.90	0.043
Current NSAID Usage	0.89	0.28, 2.77	0.839	0.51	0.16, 1.65	0.261
Current Immunosuppressive Usage	0.83	0.24, 2.85	0.764	2.11	0.72, 6.13	0.172
Patient Findings						
Clinical Signs of UTI	1.81	0.62, 5.31	0.276	0.46	0.19, 1.26	0.131
Recurrent UTI (Current)	1.17	0.38, 3.58	0.779	3.88	1.48, 10.18	0.006
Recurrent UTI (History)	5.09	1.11, 23.48	0.037	0.82	0.31, 2.23	0.704
Comorbidities	1.17	0.56, 2.45	0.685	1.23	0.64, 2.38	0.54
Pyelonephritis	.	.	0.58	0.78	0.08, 7.33	0.829

Measures of association between patient risk factors and the UPEC isolates capacity for any or strong biofilm formation. Odds ratios, 95% confidence intervals and P-values are provided.

### Multiple drug exposures increase the odds of strong biofilm formation

Approximately 85% (88/104) of the UPEC were from dogs with medical records that allowed a 30-day history review. Among those 88 patients, 43 (48.9%) had been exposed to at least one antibiotic in the past month. More detailed examination of the history identified 43 samples that had adequate history to accurately determine if patients had received more than one antibiotic in the past 30 days. Of these patients, 14 (32.6%) had received more than one antibiotic in the past 30 days. While biofilm formation was not associated with a 30-day history of antibiotic exposure (P > 0.05), isolates from dogs exposed to more than one antibiotic in the past 30 days had 4.7 times the odds of forming a strong biofilm compared to those that were exposed to only one antibiotic (95% CI: 1.05, 20.9, P = 0.043) (Table 1).

### Reduced susceptibility to antibiotics is negatively associated with biofilm production

**Fig 2:**
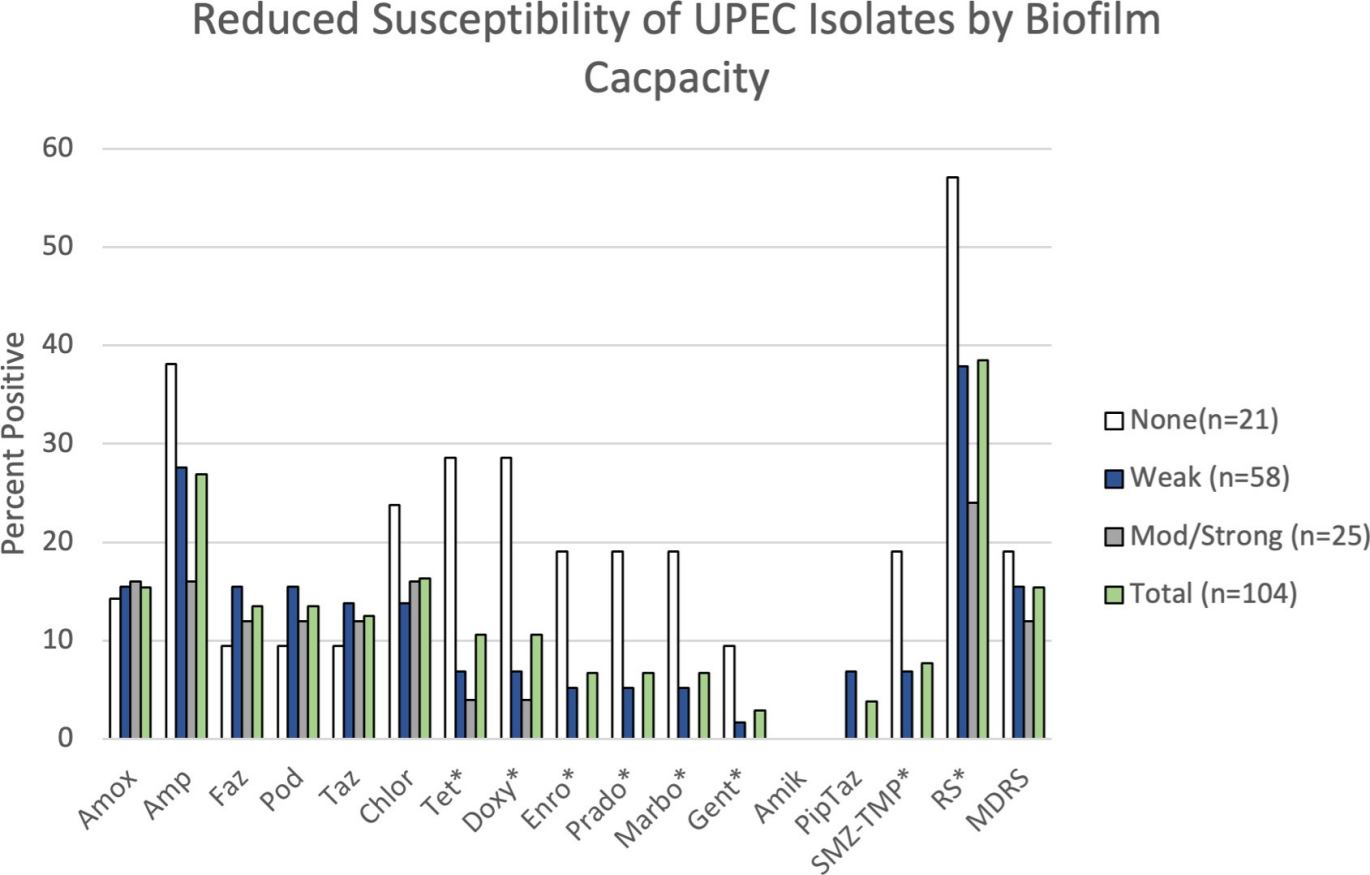
Phenotypic reduced susceptibility of UPEC isolates to 15 antimicrobials. Percentage of isolates with reduced susceptibility (RS) to the 15 antimicrobials tested stratified by biofilm capacity. Asterisk (*) indicate RS phenotypes that were negatively associated with biofilm formation (P<0.10) prior to correcting for multiple comparisons. Amox = Amoxicillin-clavulanic acid; Amp = Ampicillin; Faz = Cefazolin; Pod = Cefpodoxime; Taz = Ceftazidime; Chlor = Chloramphenicol; Tet = Tetracycline; Doxy = Doxycycline; Enro = Enrofloxacin; Marbo = Marbofloxacin; Prado = Pradofloxacin; Gent = Gentamicin; Amik = Amikacin; PipTaz = Piperacillin Tazobactam; SMZ-TMP = Trimethoprim Sulfamethoxazole; RS = Reduced susceptibility; MDRS = Multidrug reduced susceptibility.

Phenotypic reduced susceptibility (RS) to at least one antimicrobial drug was present in 40 (38.5%) of the 104 isolates collected (Fig 2). RS was most prevalent against ampicillin (26%, 27/104), followed by chloramphenicol (17.3%, 18/104) and amoxicillin-clavulanic acid (14.4%, 15/104). All of the isolates were susceptible to imipenem, a drug of last resort. Phenotypic RS decreased the odds of developing a biofilm (OR: 0.34, 95% CI: 0.13, 0.89; P = 0.029). Similarly, isolates with RS were at decreased odds of forming a strong biofilm, but this finding was of marginal statistical significance (OR: 0.42, 95% CI: 0.15, 1.16, P = 0.094). A total of 15 isolates had multidrug reduced susceptibility (14.4%) which was not associated with biofilm formation (Any: P = 0.573; Strong: P = 0.693). When individual RS phenotypes were examined, RS to tetracyclines (P = 0.008), fluoroquinolones (P = 0.029), trimethoprim-sulfamethoxazole (P = 0.052) and gentamicin (P = 0.094) were all negatively associated with biofilm formation (Fig 2). However, after correcting for multiple comparisons, none of the specific resistance phenotypes were associated with biofilm production.

### The acquired resistome is negatively associated with biofilm production

**Fig 3:**
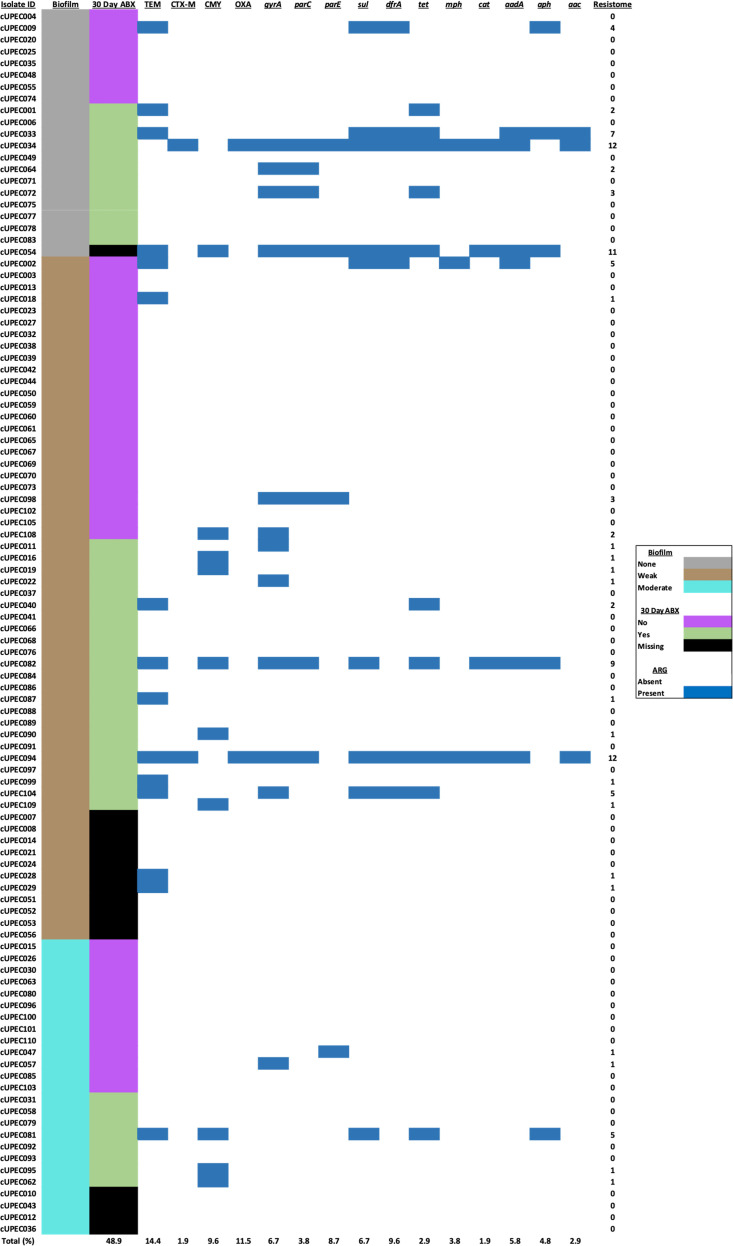
The acquired resistome profile of the 104 canine UPEC isolates. The resistome profiles of UPEC isolates stratified by biofilm formation and 30-day history of antibiotic exposure. Sample prevalence of dogs exposed to antibiotics and resistance genes is presented at the bottom of the figure. The cumulative number of acquired resistance genes in the resistome of each isolate is presented in the furthest right column.

The acquired resistome varied widely among UPEC with a mean of 0.95 genes per isolate (SD: 2.38), a median of 0, and a range of 0 to 14 genes (Fig 3). A comparison of resistance gene and biofilm capacity showed that those isolates that formed any type of biofilm were 64% less likely to harbor resistance genes compared to those that did not form biofilms (IRR: 0.36; 95% CI: 0.24, 0.53; P < 0.001). Moreover, strong biofilm producers were 68% less likely to have resistance genes (IRR: 0.32; 95% CI: 0.16, 0.63; P = 0.001) compared to weak and non-biofilm producers. Isolates that carried an allele of *tet*, *dfrA*, *or aph* or the *parC* gene were at decreased odds of producing a biofilm (P<0.05). An additional three genes–*sul*, *aac*, and *aadA–*were negatively associated with biofilm formation. However, these associations were present at marginal statistical significance (P < 0.1) (Fig 3). After controlling for multiple comparisons, none of the resistance genes were significantly associated with biofilm capacity.

### Co-occurrence of unique virulence genes predicts biofilm formation

**Fig 4:**
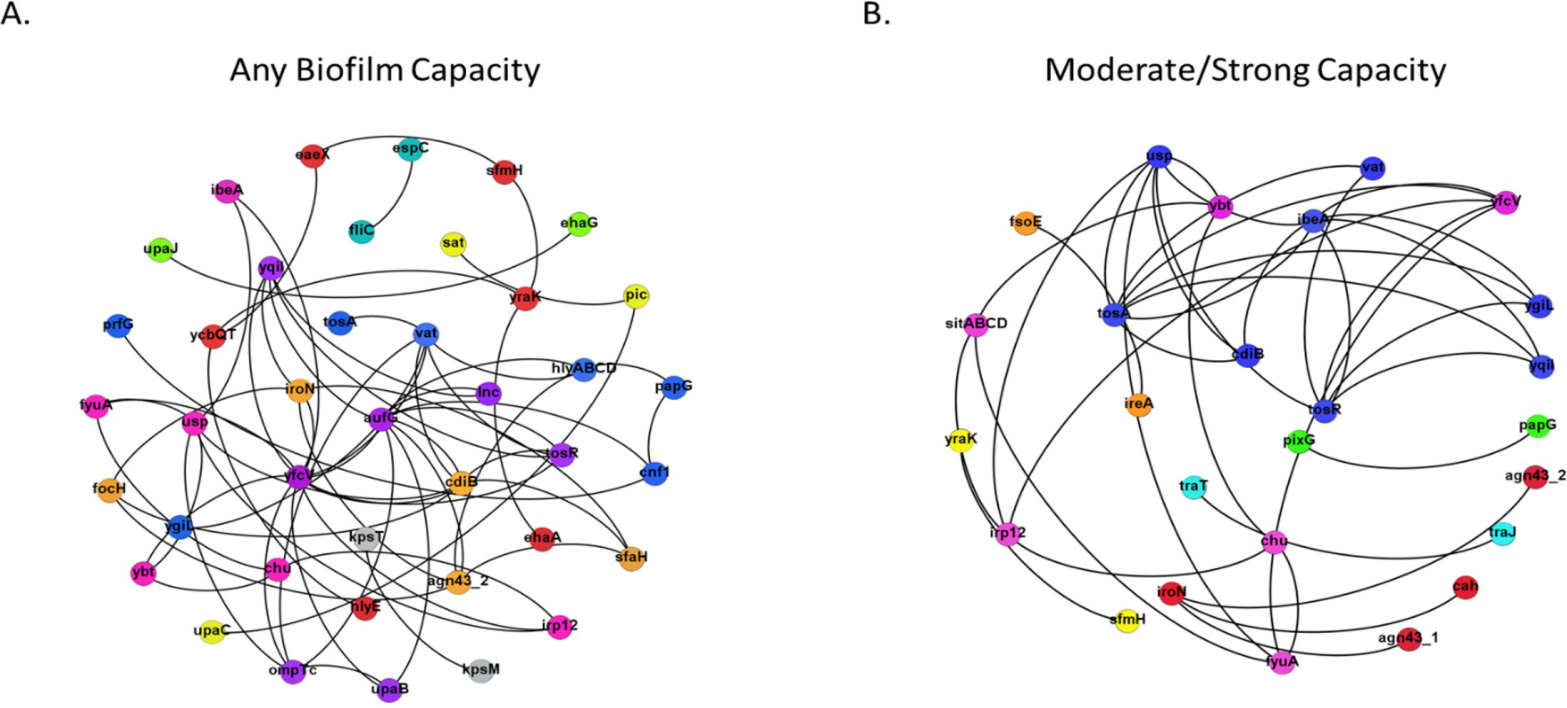
Co-occurrence networks of the unique virulence gene relationships among different levels of biofilm production. (A) Virulence co-occurrence network of unique gene relationships among isolates forming any type of biofilm. Only significant co-occurrences with a Spearman’s rho ≥0.6 are shown. (B) Virulence co-occurrence network of unique gene relationships among isolates forming moderate/strong biofilms. Only significant co-occurrences with a Spearman’s rho ≥0.6 are shown. Nodes are color coded to represent virulotype clusters based on community partitioning analysis.

We characterized the virulome using 82 virulence genes identified as significant to ExPEC or UPEC pathogens. Isolates that could form a biofilm had a slightly higher likelihood of harboring more virulence genes than non-biofilm producers (IRR: 1.07; 95% CI: 0.99, 1.15; P = 0.094). There was no difference in the number of virulence genes between strong biofilm producers when compared to weak and non-biofilm producers (P = 0.481). Despite this marginal association of the virulome to biofilm production, none of the virulence genes identified were associated with biofilm production (P > 0.05). Nine unique virulotypes were identified among isolates that formed any type of biofilm (Fig 4). However, none of these virulotypes predicted biofilm formation. Conversely, seven unique virulotypes were identified among moderate/strong biofilm producers. Isolates that carried the *ibeA-usp-vat-ygiL-cdiB-yqiI-tosA-tosR* virulotype were at 3.13 times the odds of forming a moderate/strong biofilm compared to isolates that did not harbor this virulence genotype. An analysis of gene pairs within this virulotype identified *ibeA-tosA* (OR: 3.42; 95% CI: 1.34, 8.75; P = 0.01) and *ibeA-tosR* (OR: 2.64; 95% CI: 1.05, 6.64, P = 0.039) as predictors of isolates with a moderate/strong biofilm phenotype.

### Isolates from repeat UTI have variable biofilm production

**Fig 5:**
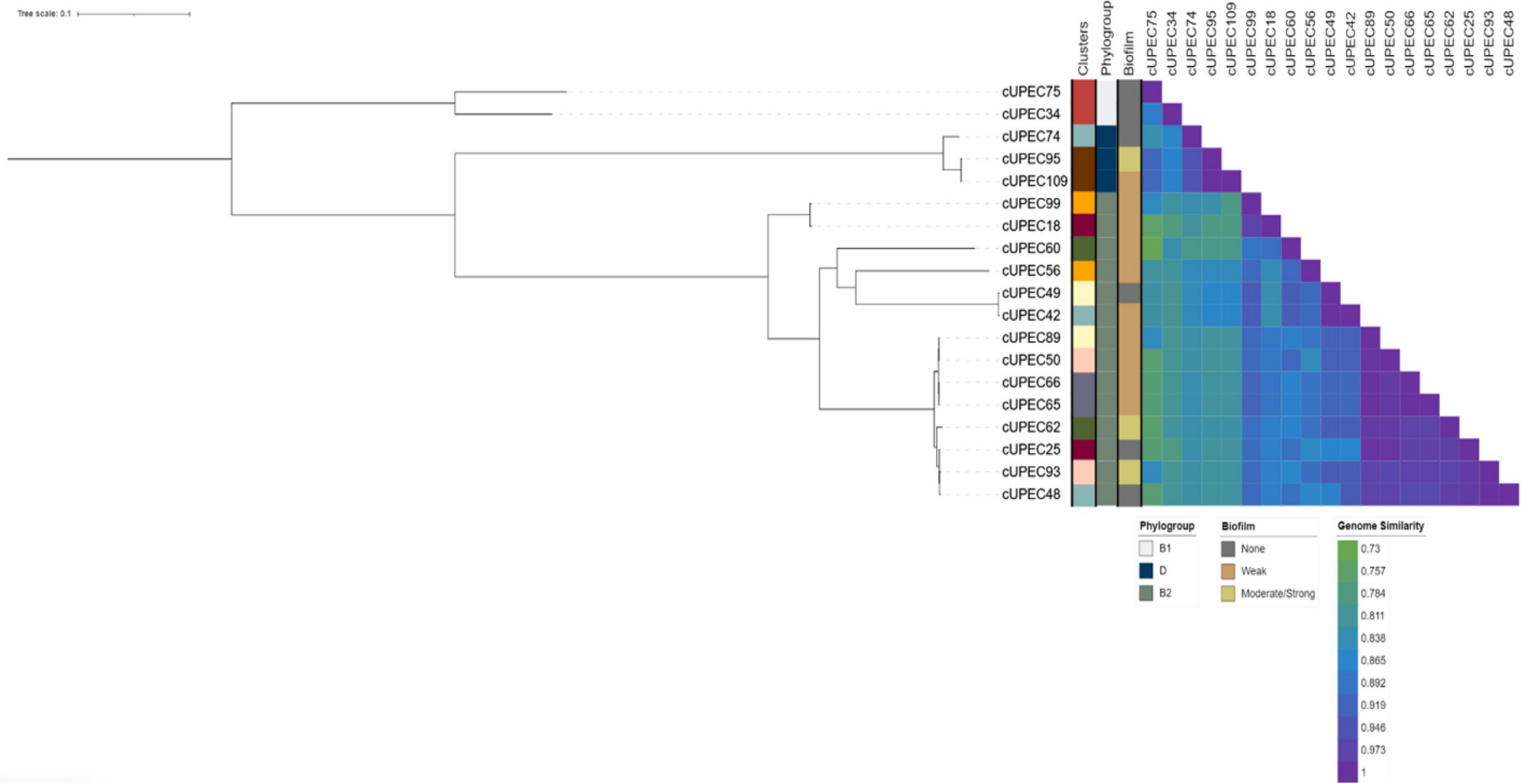
Maximum-likelihood tree of isolates from dogs that submitted two or more UPEC during the study period. Maximum likelihood phylogenetic tree of core SNP of 19 separate UPEC isolates. Metadata of the samples are present to the left of the tree including within-patient isolate clusters, isolate phylogroup, biofilm capacity and a heat map of between-isolate whole genome similarity.

Nine dogs contributed one UPEC isolate from multiple UTIs throughout the study–eight dogs submitted two isolates and one dog submitted three isolates (Fig 5). Among these 19 samples, isolates from the same patient were frequently categorized in the same phylogroup. While each isolate was genetically unique with individual virulence signatures, five pairs of isolates had greater than 99.9% genetic similarity. Two pairs (cUPEC65-cUPEC66 and cUPEC95-cUPEC109) were recovered from the same patient. However, the other pairs (cUPEC65-cUPEC89, cUPEC66-cUPEC89 and cUPEC48-cUPEC93) were recovered from different patients across the study period. Phenotypic biofilm formation varied among genetically similar UPEC pairings. There was no association between biofilm formation and the presence of individual virulence genes, antimicrobial RS, or whether an isolate was from an initial or repeat UTI (adjusted P > 0.05). Conversely, when initial *E*. *coli* isolates were compared to isolates from repeat UTIs, later isolates were at greater risk of harboring more resistance genes (IRR: 2.6; 95% CI: 0.58, 4.63; P = 0.012) and a lower quantitative virulome (IRR: 0.15; 95% CI: 0.002, 0.29; P = 0.046).

## Discussion

Here we characterized the capacity of *E*. *coli* isolated from canine UTI to form *in vitro* biofilms and observed that nearly 80% can form at least a weak biofilm. These findings suggest that *E*. *coli* isolated from canines have a slightly higher capacity to form biofilms compared to current estimates of 45–70% among uropathogenic bacteria in humans [32, 33]. While our findings show higher percentages of biofilm formation at 24 hours compared to a previous survey among canines, our prevalence of biofilm formation aligned with a previous study of urinary associated *E*. *coli* using a similar methodology [13, 14].

The isolate population consisted of diverse sequence types that primarily composed three phylogroups, B2, B1 and D. This phylogroup distribution is similar to previous reports and likely reflects the pathogenic potential of certain strains as phylogroup B2 is considered most uropathogenic, followed by phylogroup D [34]. Phylogroup B1 is considered a commensal group that can occasionally colonize the urinary tract and cause UTIs [35]. The lack of difference in biofilm capacity between phylogroups and ST is expected, as biofilm formation is used by commensals and pathogens alike to colonize advantageous niches and respond to environmental cues. However, the variability in biofilm formation within phylogroup could be associated with horizontal gene transfer as biofilm are often associated with mobile genetic elements. Our results disagree with the previous observation that isolates from ST372, a common ST among canines, form strong biofilms while ST131, a common human ST, was associated with weak or no biofilm formation [14, 36].

Patient factors like comorbidities and immune response influence the strains, virulotype and disease course of UPEC UTI. These factors may influence and select for strains with varying degrees of biofilm formation [37, 38]. However, our findings show that basic patient information, including comorbidity status, is not associated with biofilm capacity. Similar relationships between biofilm capacity and canine patient factors have been previously reported [13]. In addition, human patients with diabetes were infected with UPEC strains that did not differ in their biofilm capacity compared to non-diabetic UPEC UTI [39]. However, our study found that patients with a chronic history of UTI or that were actively in a recurrent state were more likely to harbor a biofilm forming isolate, a finding not previously reported in dogs. Biofilm formation is thought to contribute to recalcitrant, chronic UTI in humans. Women with a biofilm forming UPEC UTI are at greater odds of developing recurrent UTI [10]. Our findings provide evidence for a similar relationship in canine patients.

*E*. *coli* frequently form biofilms in the context of antimicrobial exposure to resist the oxidative stress and damage caused by some antimicrobials [40]. Here we show that antibiotic use within 30 days prior to infection did not influence the biofilm capacity of the colonizing UPEC strain. However, if the patient was exposed to more than one antibiotic over the course of 30 days prior to UTI the infecting UPEC strains were at greater odds of forming a strong biofilm. The physical properties of the extracellular matrix covering biofilms creates an environment that inhibits permeation of antimicrobials to the underlying bacteria [41]. In addition, bacteria can enter a stationary or dormant growth phenotype, which can increase their resistance to antibiotics targeting growth/reproduction/nutritional pathways [40]. Moreover, biofilms can be comprised of multiple heterogeneous subpopulations that support the generation of resistant strains and serves as hotspots for horizontal transfer of resistance genes [40, 42]. All these factors may be involved in the capacity of multidrug exposure to elicit a biofilm phenotype.

Isolates with reduced susceptibility to antimicrobials were at lesser odds of forming biofilms, however this association was not apparent with multidrug-reduced susceptibility phenotypes. Specific RS phenotypes associated with decreased biofilm production were tetracyclines, fluoroquinolones, trimethoprim-sulfamethoxazole and aminoglycosides. We also corroborated the association of reduced susceptibility and biofilm formation with resistomics data. Phenotypic antimicrobial resistance, including those to fluoroquinolones and trimethoprim-sulfamethoxazole, has been negatively associated with biofilm formation [12, 14]. UPEC with biofilm capacity may not require costly antimicrobial resistance genes because the extracellular matrix serves as a barrier to antimicrobial diffusion. However, other studies in canines found that fluoroquinolone resistance was positively associated with biofilm formation, a common finding among the human literature [10, 43, 44]. This suggests that some strains have adapted the capacity to harbor antimicrobial resistance genes and biofilm formation genes despite their duplicity in AMR. UPEC from canine sources can harbor antimicrobial resistance genes, including fluoroquinolone resistance genes, that are linked to virulence genes associated with biofilm formation in rat models [45]. Application of whole genome surveys with phenotypic findings is required to further understand the relationship of antimicrobial resistance and biofilm formation.

While marginal increases in the overall virulome increased with biofilm forming capacity, independent virulence genes were not associated with biofilm phenotype. Moreover, genetically similar organisms differed in biofilm capacity. However, even genetically related organisms maintained heterogenous virulotypes. Together, these findings support previous observations that *E*. *coli*, especially uropathogenic strains, utilize a “mix-and-match” formula to form biofilms. In part, this redundancy and heterogeneity complicates attempts to accurately predict biofilm phenotypes among UPEC isolates [12]. This is apparent in the human and canine literature where independent virulence genes including adhesins, iron acquisition systems and toxins are inconsistently associated with greater biofilm capacity [13, 14, 46, 47]. Co-occurrence and community analysis offers a unique approach to identify virulence gene pairs or communities, instead of single genes, that are associated with isolates forming moderate/strong biofilms. Using this approach, two gene combinations (*ibeA-tosA* and *ibeA-tosR*) were associated with biofilm capacity. The *tos* operon mediates fimbrial adhesins necessary for adhesion and biofilm formation while *ibeA* is an invasin that promotes biofilm adhesion by interacting with surface epithelium [48, 49]. Approaches that identify gene communities and pairings may offer a more sensitive approach at predicting biofilm formation, but further studies are required.

Future studies using broad and targeted genomic surveys will help to identify novel UPEC virulence genes that contribute to biofilm formation. Large-scale genomic approaches that attempt to associate mobile genetic elements with biofilm formation will also provide insight on how horizontal gene transfer impact biofilm phenotype. RNA sequencing of UPEC under a variety of environmental conditions, such as those that include antibiotic selective pressure, *and in* vivo states will further our understanding of the complex pathogen-environment and host-pathogen interactions that underlie biofilm formation.

## Conclusions

Here we identified patient and pathogen factors that are related to biofilm formation *in vitro*. Dogs with a history or active recurrent UTI that receive multiple rounds of antibiotics are at increased odds of being infected with a biofilm forming isolate. However, urinary *E*. *coli* with phenotypic resistance were negatively associated with biofilm formation. We posit that the use of antimicrobials can select for two types of populations, one that resist antibiotics as planktonic, free-living species via acquired antimicrobial resistance genes and a separate population that resist antibiotic therapy by establishing mature, slow growing biofilms. In both cases these pathogens can cause recurrent infections that lead to more selective pressure from antibiotic use. This environment can create a vicious cycle of UTIs and antibiotic use that contributes to recurrence. While virulence genes are necessary for colonization, infection, and biofilm formation, the diverse and redundant virulome of UPEC and urinary-associated *E*. *coli* creates numerous genetic combinations that all reach a similar phenotypic endpoint.

## Supporting information

S1 File Study metadata including biofilm outcomes, antibiotic susceptibility results, virulence gene matrix, and all other epidemiologic data. (XLSX)

## References

[1] Johnson JR, Russo TA. Molecular epidemiology of extraintestinal pathogenic *Escherichia coli*. EcoSal Plus. 2018;8(1). doi: 10.1128/ecosalplus.ESP-0004-2017 29667573 PMC11575673

[2] Croxen MA and Finlay BB. Molecular mechanisms of *Escherichia coli* pathogenicity. Nat Rev Micro. 2010;8: 26–38. 10.1038/nrmicro2265 19966814

[3] Thompson MF, Litster AL, Platell JL, Trott DJ. Canine bacterial urinary tract infections: new developments in old pathogens. Vet J. 2011;190(1):22–7. doi: 10.1016/j.tvjl.2010.11.013 21239193

[4] Ling GV. Therapeutic strategies involving antimicrobial treatment of the canine urinary tract. J Am Vet Med Assoc. 1984;185(10):1162–4. 6392247

[5] Ball KR, Rubin JE, Chirino-Trejo M, Dowling PM. Antimicrobial resistance and prevalence of canine uropathogens at the Western College of Veterinary Medicine Veterinary Teaching Hospital, 2002–2007. Can Vet J. 2008;49(10):985–90. 19119366 PMC2553511

[6] Wong C, Epstein SE, Westropp JL. Antimicrobial Susceptibility Patterns in Urinary Tract Infections in Dogs (2010–2013). J Vet Intern Med. 2015;29(4):1045–52. doi: 10.1111/jvim.13571 26133165 PMC4895361

[7] Flores-Mireles AL, Walker JN, Caparon M, Hultgren SJ. Urinary tract infections: epidemiology, mechanisms of infection and treatment options. Nat Rev Microbiol. 2015;13(5):269–84. doi: 10.1038/nrmicro3432 25853778 PMC4457377

[8] Landini P, Antoniani D, Burgess JG, Nijland R. Molecular mechanisms of compounds affecting bacterial biofilm formation and dispersal. Appl Microbiol Biotechnol. 2010;86(3):813–23. doi: 10.1007/s00253-010-2468-8 20165945

[9] Costerton JW, Stewart PS, Greenberg EP. Bacterial biofilms: a common cause of persistent infections. Science. 1999;284(5418):1318–22. doi: 10.1126/science.284.5418.1318 10334980

[10] Soto SM, Smithson A, Horcajada JP, Martinez JA, Mensa JP, Vila J. Implication of biofilm formation in the persistence of urinary tract infection caused by uropathogenic Escherichia coli. Clin Microbiol Infect. 2006;12(10):1034–6. doi: 10.1111/j.1469-0691.2006.01543.x 16961644

[11] Prüss BM, Besemann C, Denton A, Wolfe AJ. A complex transcription network controls the early stages of biofilm development by Escherichia coli. J Bacteriol. 2006;188(11):3731–9. doi: 10.1128/JB.01780-05 16707665 PMC1482888

[12] Reisner A, Haagensen JA, Schembri MA, Zechner EL, Molin S. Development and maturation of Escherichia coli K-12 biofilms. Mol Microbiol. 2003;48(4):933–46. doi: 10.1046/j.1365-2958.2003.03490.x 12753187

[13] Kern ZT, Jacob ME, Gilbertie JM, Vaden SL, Lyle SK. Characteristics of Dogs with Biofilm-Forming Escherichia Coli Urinary Tract Infections. J Vet Intern Med. 2018;32(5):1645–51. doi: 10.1111/jvim.15231 30084122 PMC6189388

[14] Gilbertie JM, Levent G, Norman KN, Vinasco J, Scott HM, Jacob ME. Comprehensive phenotypic and genotypic characterization and comparison of virulence, biofilm, and antimicrobial resistance in urinary Escherichia coli isolated from canines. Vet Microbiol. 2020;249:108822. doi: 10.1016/j.vetmic.2020.108822 32937249

[15] Behzadi P, Urbán E, Gajdács M. Association between Biofilm-Production and Antibiotic Resistance in Uropathogenic. Diseases. 2020;8(2). doi: 10.3390/diseases8020017 PMC7348726 32517335

[16] Weese JS, Blondeau JM, Boothe D, Breitschwerdt EB, Guardabassi L, Hillier A, et al. Antimicrobial use guidelines for treatment of urinary tract disease in dogs and cats: antimicrobial guidelines working group of the international society for companion animal infectious diseases. Vet Med Int. 2011;2011:263768. doi: 10.4061/2011/263768 21776346 PMC3134992

[17] Stępień-Pyśniak D, Hauschild T, Kosikowska U, Dec M, Urban-Chmiel R. Biofilm formation capacity and presence of virulence factors among commensal Enterococcus spp. from wild birds. Sci Rep. 2019;9(1):11204. doi: 10.1038/s41598-019-47602-w 31371744 PMC6671946

[18] National Committee for Clinical Laboratory Standards. Performance standards for antimicrobial disk and dilution susceptibility testing for bacteria isolated from animals: VET10S. 5th edition. Wayne (PA): Clinical and Laboratory Standards Institute; 2020.

[19] Magiorakos AP, Srinivasan A, Carey RB, Carmeli Y, Falagas ME, Giske CG, et al. Multidrug-resistant, extensively drug-resistant and pandrug-resistant bacteria: an international expert proposal for interim standard definitions for acquired resistance. Clin Microbiol Infect. 2012;18(3):268–81. doi: 10.1111/j.1469-0691.2011.03570.x 21793988

[20] Krueger F. Trim Galore: A wrapper tool around Cutadapt and FastQC to consistently apply quality and adapter trimming to FastQ files. 2019 Nov 19 [Cited 2021 Aug 15). Available from: http://www.bioinformatics.babraham.ac.uk/projects/trim_galore/.

[21] Andrews S. FastQC: A quality control tool for high throughput sequence data. 2019 Jan 08 [Cited 2021 Aug 15]. Available from: http://www.bioinformatics.babraham.ac.uk/projects/fastqc/.

[22] Wick RR, Judd LM, Gorrie CL, Holt KE. Unicycler: Resolving bacterial genome assemblies from short and long sequencing reads. PLoS Comput Biol. 2017;13(6):e1005595. doi: 10.1371/journal.pcbi.1005595 28594827 PMC5481147

[23] Seemann T. Prokka: rapid prokaryotic genome annotation. Bioinformatics. 2014;30(14):2068–9. doi: 10.1093/bioinformatics/btu153 24642063

[24] Bortolaia V, Kaas RS, Ruppe E, Roberts MC, Schwarz S, Cattoir V, et al. ResFinder 4.0 for predictions of phenotypes from genotypes. J Antimicrob Chemother. 2020;75(12):3491–500. doi: 10.1093/jac/dkaa345 32780112 PMC7662176

[25] Leimbach A. ecoli_VF_collection: V.0.1. Zenodo. 2016. Available from: 10.5281/zenodo.56686.

[26] Beghain J, Bridier-Nahmias A, Le Nagard H, Denamur E, Clermont O. ClermonTyping: an easy-to-use and accurate in silico method for Escherichia genus strain phylotyping. Microb Genom. 2018;4(7). doi: 10.1099/mgen.0.000192 29916797 PMC6113867

[27] Zhou Z, Alikhan NF, Mohamed K, Fan Y, Achtman M, Group AS. The EnteroBase user’s guide, with case studies on. Genome Res. 2020;30(1):138–52. doi: 10.1101/gr.251678.119 31809257 PMC6961584

[28] Kaas RS, Leekitcharoenphon P, Aarestrup FM, Lund O. Solving the problem of comparing whole bacterial genomes across different sequencing platforms. PLoS One. 2014;9(8):e104984. Epub 20140811. doi: 10.1371/journal.pone.0104984 25110940 PMC4128722

[29] Letunic I, Bork P. Interactive Tree Of Life (iTOL) v4: recent updates and new developments. Nucleic Acids Res. 2019;47(W1):W256–W9. doi: 10.1093/nar/gkz239 30931475 PMC6602468

[30] Csardi G, Nepusz T. The igraph software package for complex network research. InterJournal, 2006;Complex Systems: 1695. http://Igraph.sf.net.

[31] Bastian M., Heymann S., Jacomy M. Gephi: an open source software for exploring and manipulating networks. International AAAI Conference on Weblogs and Social Media. 2009.

[32] Tewawong N, Kowaboot S, Pimainog Y, Watanagul N, Thongmee T, Poovorawan Y. Distribution of phylogenetic groups, adhesin genes, biofilm formation, and antimicrobial resistance of uropathogenic. PeerJ. 2020;8:e10453. doi: 10.7717/peerj.10453 33344087 PMC7718785

[33] Soto SM, Smithson A, Martinez JA, Horcajada JP, Mensa J, Vila J. Biofilm formation in uropathogenic Escherichia coli strains: relationship with prostatitis, urovirulence factors and antimicrobial resistance. J Urol. 2007;177(1):365–8. doi: 10.1016/j.juro.2006.08.081 17162092

[34] Hutton TA, Innes GK, Harel J, Garneau P, Cucchiara A, Schifferli DM, et al. Phylogroup and virulence gene association with clinical characteristics of Escherichia coli urinary tract infections from dogs and cats. J Vet Diagn Invest. 2018;30(1):64–70. doi: 10.1177/1040638717729395 28971754 PMC5746180

[35] Baldiris-Avila R, Montes-Robledo A, Buelvas-Montes Y. Phylogenetic Classification, Biofilm-Forming Capacity, Virulence Factors, and Antimicrobial Resistance in Uropathogenic Escherichia coli (UPEC). Curr Microbiol. 2020;77(11):3361–70. doi: 10.1007/s00284-020-02173-2 32910213

[36] Surgers L, Boyd A, Girard PM, Arlet G, Decré D. Biofilm formation by ESBL-producing strains of Escherichia coli and Klebsiella pneumoniae. Int J Med Microbiol. 2019;309(1):13–8. doi: 10.1016/j.ijmm.2018.10.008 30385204

[37] Johnson JR, Porter S, Johnston B, Kuskowski MA, Spurbeck RR, Mobley HL, et al. Host Characteristics and Bacterial Traits Predict Experimental Virulence for Escherichia coli Bloodstream Isolates From Patients With Urosepsis. Open Forum Infect Dis. 2015;2(3):ofv083. doi: 10.1093/ofid/ofv083 26199950 PMC4504731

[38] Johnson JR, Moseley SL, Roberts PL, Stamm WE. Aerobactin and other virulence factor genes among strains of Escherichia coli causing urosepsis: association with patient characteristics. Infect Immun. 1988;56(2):405–12. doi: 10.1128/iai.56.2.405-412.1988 2892793 PMC259296

[39] Raya S, Belbase A, Dhakal L, Govinda Prajapati K, Baidya R, Kishor Bimali N. In-Vitro Biofilm Formation and Antimicrobial Resistance of. Biomed Res Int. 2019;2019:1474578. doi: 10.1155/2019/1474578 31641666 PMC6770373

[40] Ito A, Taniuchi A, May T, Kawata K, Okabe S. Increased antibiotic resistance of Escherichia coli in mature biofilms. Appl Environ Microbiol. 2009;75(12):4093–100. doi: 10.1128/AEM.02949-08 19376922 PMC2698376

[41] Limoli DH, Jones CJ, Wozniak DJ. Bacterial Extracellular Polysaccharides in Biofilm Formation and Function. Microbiol Spectr. 2015;3(3). doi: 10.1128/microbiolspec.MB-0011-2014 26185074 PMC4657554

[42] Madsen JS, Burmølle M, Hansen LH, Sørensen SJ. The interconnection between biofilm formation and horizontal gene transfer. FEMS Immunol Med Microbiol. 2012;65(2):183–95. doi: 10.1111/j.1574-695X.2012.00960.x 22444301

[43] Cepas V, López Y, Muñoz E, Rolo D, Ardanuy C, Martí S, et al. Relationship Between Biofilm Formation and Antimicrobial Resistance in Gram-Negative Bacteria. Microb Drug Resist. 2019;25(1):72–9. doi: 10.1089/mdr.2018.0027 30142035

[44] Oliveira M, Dias FR, Pomba C. Biofilm and fluoroquinolone resistance of canine Escherichia coli uropathogenic isolates. BMC Res Notes. 2014;7:499. doi: 10.1186/1756-0500-7-499 25099929 PMC4132243

[45] Wroblewska-Seniuk K, Selvarangan R, Hart A, Pladzyk R, Goluszko P, Jafari A, et al. Dra/AfaE adhesin of uropathogenic Dr/Afa+ Escherichia coli mediates mortality in pregnant rats. Infect Immun. 2005;73(11):7597–601. doi: 10.1128/IAI.73.11.7597-7601.2005 16239563 PMC1273835

[46] Naves P, del Prado G, Huelves L, Gracia M, Ruiz V, Blanco J, et al. Correlation between virulence factors and in vitro biofilm formation by Escherichia coli strains. Microb Pathog. 2008;45(2):86–91. Epub doi: 10.1016/j.micpath.2008.03.003 . 18486439

[47] Ejrnæs K, Stegger M, Reisner A, Ferry S, Monsen T, Holm SE, et al. Characteristics of *Escherichia coli* causing persistence or relapse of urinary tract infections: phylogenetic groups, virulence factors and biofilm formation. Virulence. 2011;2: 528–37. doi: 10.4161/viru.2.6.18189 22030858

[48] Luterbach CL, Forsyth VS, Engstrom MD, Mobley HLT. TosR-Mediated Regulation of Adhesins and Biofilm Formation in Uropathogenic Escherichia coli. mSphere. 2018;3(3). Epub 20180516. doi: 10.1128/mSphere.00222-18 29769381 PMC5956150

[49] Wang S, Niu C, Shi Z, Xia Y, Yaqoob M, Dai J, et al. Effects of ibeA deletion on virulence and biofilm formation of avian pathogenic Escherichia coli. Infect Immun. 2011;79(1):279–87. Epub 20101025. doi: 10.1128/IAI.00821-10 20974831 PMC3019902

